# Supplementary material for: Resolving the full spectrum of human genome variation using Linked-Reads
Source: Genome Res. 2019 Apr;29(4):635–45. doi: 10.1101/gr.234443.118 (PMC6442396; doi:10.1101/gr.234443.118)
Supplement: Supplemental Material [file supp_gr.234443.118_Supplemental_longranger-2.2.zip › longranger-2.2.tar.bz2!/lr2.2-public/lib/stan/cmdstan/stan/doc/api/html/struct_eigen_1_1internal_1_1significant__decimals__default__impl_3_01stan_1_1agrad_1_1var_00_01false_01_4.html]

Stan: Eigen::internal::significant\_decimals\_default\_impl< stan::agrad::var, false > Struct Template Reference


|  |  |
| --- | --- |
|  | Stan  2.5.0  probability, sampling & optimization |


- Main Page
- Related Pages
- Namespaces
- Classes
- Files

- Class List
- Class Index
- Class Hierarchy
- Class Members

All Classes Namespaces Files Functions Variables Typedefs Enumerations Enumerator Friends Macros Pages

- Eigen
- internal
- significant\_decimals\_default\_impl< stan::agrad::var, false >

Static Public Member Functions |
List of all members

Eigen::internal::significant\_decimals\_default\_impl< stan::agrad::var, false > Struct Template Reference

Implemented this for printing to stream.
More...

`#include <Eigen_NumTraits.hpp>`

|  |  |
| --- | --- |
| Static Public Member Functions | |
| static int | run () |
|  | |

## Detailed Description

### template<> struct Eigen::internal::significant\_decimals\_default\_impl< stan::agrad::var, false >

Implemented this for printing to stream.

Definition at line 145 of file Eigen\_NumTraits.hpp.

## Member Function Documentation

|  |  |  |  |  |
| --- | --- | --- | --- | --- |
| static int Eigen::internal::significant\_decimals\_default\_impl< stan::agrad::var, false >::run | ( |  | ) |  |

inlinestatic

Definition at line 147 of file Eigen\_NumTraits.hpp.

---

The documentation for this struct was generated from the following file:

- src/stan/agrad/rev/matrix/Eigen\_NumTraits.hpp


---

[ Stan Home Page ]

*© 2011–2014,
Stan Development Team.*
